# Nuclear survivin as a biomarker for non-small-cell lung cancer

**DOI:** 10.1038/sj.bjc.6602027

**Published:** 2004-07-13

**Authors:** B Lu, A Gonzalez, P P Massion, Y Shyr, B Shaktour, D P Carbone, D E Hallahan

**Affiliations:** 1 Department of Radiation Oncology, Vanderbilt Ingram, Cancer Center, Vanderbilt University School of Medicine, Nashville, TN 37232, USA; 2 Department of Pathology, Vanderbilt Ingram, Cancer Center, Vanderbilt University School of Medicine, Nashville, TN 37232, USA; 3 Department of Medicine, Vanderbilt Ingram, Cancer Center, Vanderbilt University School of Medicine, Nashville, TN 37232, USA; 4 Department of Preventive Medicine, Vanderbilt Ingram, Cancer Center, Vanderbilt University School of Medicine, Nashville, TN 37232, USA

**Keywords:** survivin, lung cancer, nucleus, tissue array, mitosis, apoptosis

## Abstract

Survivin inhibits apoptosis and promotes mitosis. We determined whether nuclear or cytoplasmic localisation of survivin predicts survival of 48 patients with resected non-small-cell lung cancer (NSCLC). Patients with nuclear staining of survivin had significantly worse survival (relative risk: 3.9, *P*=0.02). Therefore, survivin may be a biomarker for NSCLC.

Survivin was initially identified as an inhibitor of apoptosis. Its expression is undetectable in most terminally differentiated normal tissues, but is at high levels in various malignancies as well as embryonic and fetal tissues (Salvesen and Duckett, 2002). Survivin expression is upregulated in all phases of cell cycle, and the cancer-specific activity of survivin promoter was detected both *in vivo* and *in vitro* (Bao *et al*, 2002). Deletion of survivin resulted in a catastrophic defect of microtubule assembly, with absence of mitotic spindles, disorganised tubulin aggregates and multinucleation, in the survivin knockout mice (Uren *et al*, 2000). Colocalisation of survivin with Aurora-B and the inner centromere protein (INCENP) suggests that these proteins interact throughout mitosis and are essential for chromosome condensation and segregation as well as the completion of cytokinesis (Adams *et al*, 2001). Several studies have shown that survivin is a prognostic indicator for poor survival in several malignancies (Adida *et al*, 2000; Chakravarti *et al*, 2002; Kennedy *et al*, 2003; Trieb *et al*, 2003). Survivin protein level examined by immunostaining was associated with vascular invasion and poor survival (Ikehara *et al*, 2002). However, the predictive value of survivin mRNA examined by RT–PCR is contradictory between two independent studies (Monzo *et al*, 1999, Falleni *et al*, 2003). Survivin staining was found in both nucleus and cytoplasm of non-small-cell lung cancer (NSCLC) (Falleni *et al*, 2003). Nuclear survivin rather than the cytoplasmic staining was shown to be predictive of poor survival in patients with oesophageal cancers (Grabowski *et al*, 2003). Since survivin has both nuclear and cytoplasmic targets and is involved in regulation of mitosis and apoptosis, we determined the predictive value of nuclear *vs* cytoplasmic staining of survivin among patients with resected NSCLC.

## METHODS

### Patients

Archived tissue blocks from 1999 to 2002 were retrieved from the files of Vanderbilt University and the Nashville Veterans Affairs Medical Center pathology departments, according to the approved IRB protocol (010178). For all tissue blocks, the H&E-stained sections were reviewed by two pathologists who specialise in lung cancer. Table 1

**Table 1:** Association of clinical variables and survivin staining with overall survival by the log-rank test

**Variable**	**Number of patients**	**Median survival (days)**	**95% CI of median survival**	***P*-value**
*Age (years)*				
<60	22	458	(294, NR)	NS
⩾60	26	NR	(133, NR)	
*Sex*				
Female	18	NR	(452, NR)	NS
Male	28	NR	(292, NR)	
*Grade*				
I	5	NR	(NR, NR)	NS
II	20	858	(179, NR)	
III	14	292	(122, NR)	
*T-stage*				
1	25	NR	(NR, NR)	<.0001
2	19	458	(292, NR)	
3	0			
4	4	64	(12, 204)	
*Node stage*				
0	36	NR	(452, NR)	NS
1 and 2	12	235	(122, NR)	
*Nuclear survivin*				
Neg	40	NR	(440, NR)	0.0116
Pos	8	248	(68, 458)	
*Cytoplasmic survivin*				
Neg	18	858	(235, NR)	NS
Pos	30	469	(292, NR)	

NR=not reached; NS=not significant.

summarises the clinical and molecular characteristics of 48 patients with NSCLC. Patients underwent surgical resection with hilar and mediastinal lymph node sampling. None of these patients received neoadjuvant chemotherapy or radiotherapy. Clinical data was obtained from the tumour registry and hospital charts at the Vanderbilt Medical Center.

### Immunohistochemistry

Paraffin-embedded material was available in a set of 48 individual tumours for evaluation of nuclear and cytoplasmic staining of survivin. These studies were carried out using a standard avidin–biotin–peroxidase complex technique, with a mouse monoclonal antibody against survivin (Santa Cruz Biotech, Santa Cruz, California, sc-17779). Three separate sections from each case were examined by the immunohistochemistry method. Staining was assessed in 5–10 high-powered fields at × 400 magnification. Survivin immunoreactivity was evaluated semiquantitatively based on the intensity of staining. It was scored as: 1+ (weak); 2+ (moderate); and 3+ (intense). Cases with no or weak staining were considered negative, whereas those with moderate to intense staining were considered as positive. The highest score among the three tissue sections was entered for statistical analyses.

### Statistical analysis

For lifetime data analyses, the possible risk factors, for example, nuclear survivin, were compared for survival with the Kaplan–Meier estimates and log-rank tests. The proportional hazards model was used for adjusted tests of significance and estimates of odds ratios. The unadjusted and adjusted 95% confidence intervals (CI) of survival were calculated and reported for univariate and multivariate statistical models. All tests of significance were two sided, and differences were considered statistically significant when *P*-value was <0.05. SAS version 8.2 and S-Plus 6 were used for all analyses.

## RESULTS

### Cytoplasmic and nuclear staining of survivin in the tissue samples of NSCLC

**Figure 1:**
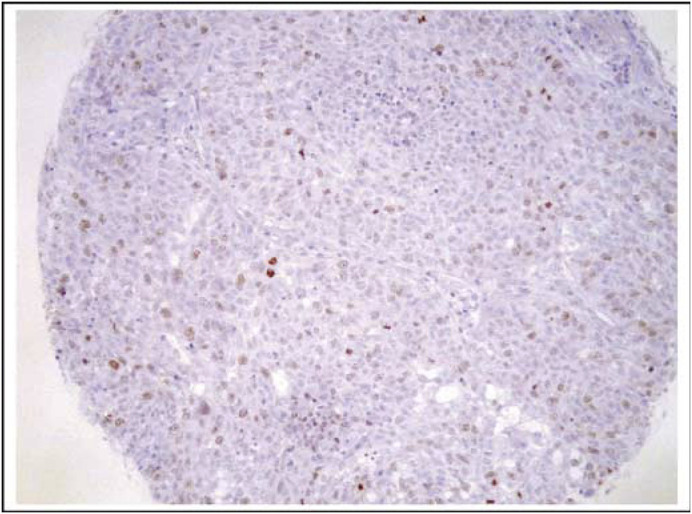
Cytoplasmic and nuclear staining of NSCLC: one of the tissue cores containing NSCLC was stained with the mouse monoclonal antibody against survivin, sc-17779.

We have performed immunohistochemistry analyses on tissue cores containing NSCLCs from 48 patients who underwent surgical resections at Vanderbilt University Medical Center from 1999 to 2002. Figure 1

shows a tissue core stained with the sc-17779 mouse monoclonal antibody. As shown, prominent nuclear staining was observed. A total of 32 cases (67%) were scored as positive for nuclear staining, whereas 39 cases (83%) were scored as positive for cytoplasmic staining. A totl of 19 cases (44%) had positive staining in both cytoplasm and nucleus.

### Nuclear survivin is associated with poor survival in resected NSCLC

**Figure 2:**
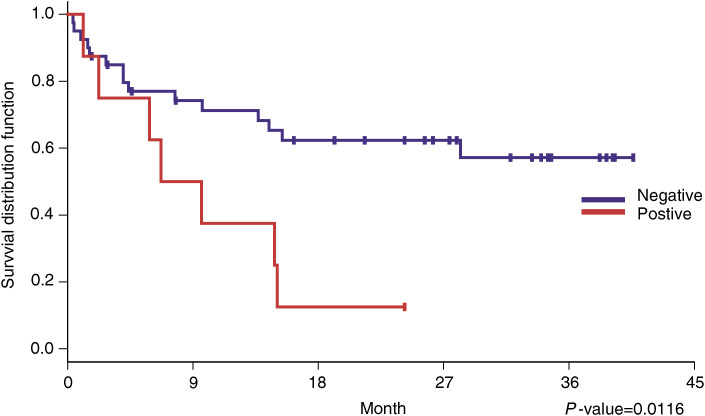
Overall survival was significantly worse among NSCLC patients with positive nuclear survivin staining. The Kaplan–Meier survival curve (*P*=0.01 by univariate analysis) is shown.

In order to determine whether immunohistochemistry of survivin staining has any prognostic value, we examined the association between nuclear or cytoplasmic staining of survivin with overall survival among the 48 patients with resected NSCLC. No difference in survival was detected when the data was analysed by patients’ age or sex, tumour histology or grade, or lymph node status. However, T stage was significantly associated with poor survival as shown in Table 1. As for survivin staining, nuclear survivin positivity was significantly associated with poor survival (*P*=0.01, median survival of 248 days, 95% CI: 68–458 days), whereas cytoplasmic staining of survivin was not, as shown in Figure 2

. This was confirmed by multivariate analyses, which demonstrated a relative risk of 3.9 in the patients with positive nuclear survivin (*P*=0.02), as shown in Table 2

**Table 2:** Multivariate analysis of nuclear presence of survivin and survival

**Variable**	**Relative risk**	**95% CI**	***P*-value**
Nuclear-survivin	3.92	1.31, 11.79	0.02
Cyto-survivin	1.20	0.42, 3.46	0.74
Age	1.22	0.47, 3.19	0.69
Gender	0.97	0.38, 2.51	0.95

.

## DISCUSSION

In this study, we have found that the nuclear presence of survivin was associated with poor survival in patients with NSCLC. Both mRNA and protein levels of survivin were shown to predict unfavourable survival in patients with resected NSCLC (Monzo *et al*, 1999; Ikehara *et al*, 2002). Since survivin has dual function in apoptosis and mitosis depending upon its cellular localisation, the predictive value of nuclear *vs* cytoplasmic staining of survivin has been investigated in a number of malignancies. When nuclear or cytoplasmic staining of survivin was scored separately, nuclear staining was linked with favourable prognosis in gastric cancer (Okada *et al*, 2001), breast cancer (Kennedy *et al*, 2003) and osteosarcoma (Trieb *et al*, 2003), whereas cytoplasmic survivin was not found to be prognostic. In contrast, a recent study in oesophageal cancers showed that nuclear survivin was associated with poor survival (Grabowski *et al*, 2003). Our results in NCSLC also demonstrated a poor prognostic value of nuclear survivin. The prognostic difference of nuclear survivin among these studies appears to be tumoor specific. Relative importance of nuclear survivin in mitosis may vary among different tumour types and may predict differently the responses to various cancer type-specific therapies, which ultimately determine the overall survival of cancer patients. Nuclear and cytoplasmic pools of survivin have their distinct roles (Fortugno *et al*, 2002). It has been shown that survivin splice variants had different subcellular localisations (Mahotka *et al*, 2002). Survivin-delta Ex3 is preferentially localised in the nucleus, whereas survivin and survivin 2B isoforms are found in the cytoplasm. However, survivin-2B is nonantiapoptotic. It is not surprising that the cytoplasmic level is not prognostic since the IHC staining is unable to discriminate the splicing variants and represents the combined level of two survivin variants with opposing effects on apoptosis. The RNA level of survivin 2B (nonantiapoptotic) detected by RT–PCR was, however, found decreased in advanced stages of renal (Mahotka *et al*, 2002) and gastric cancers (Krieg *et al*, 2002).

In the nucleus, survivin was shown to interact with aurora B kinase and INCENP, which play essential roles in chromosomal segregation during the exit of mitosis (Honda *et al*, 2003). Knockdown and inhibition of survivin resulted in multinucleated and polyploid cells, which is a phenotype of mitotic arrest (Uren *et al*, 2000). Therefore, strong expression of survivin in the nucleus may represent increased mitotic events. On the other hand, in the cytoplasm, survivin inhibits apoptosis by blocking caspase activity. So far, it has not been demonstrated that the cytoplasmic survivin alone predicts clinical outcome. This may result from that the measurement of cytoplasmic survivin includes the combined level of two functionally opposing variants. Alternatively, other antiapoptotic molecules such as bcl-2 proteins may be more important players in lung cancers. However, a dominant negative mutant of survivin, T34A, has been shown to be effective in treating xenografts of breast cancer by freeing up caspase 9 and thus promoting apoptosis (Wall *et al*, 2003). This suggests that survivin remains a viable therapeutic target in certain cancers (Altieri, 2003).

In summary, nuclear presence of survivin may be an important prognostic marker for patients with resected NSCLC. Larger population studies are needed to confirm the value of nuclear staining of survivin as a prognostic marker. Further investigation should evaluate the strategies of intervening survivin function for therapeutics in lung cancer.
